# Atypical Acute and Non-traumatic Presentation of an Indirect Carotid-Cavernous Fistula

**DOI:** 10.7759/cureus.82996

**Published:** 2025-04-25

**Authors:** Bushra Kurban, Syed Mustafa, Amer Rehman, Parviz Dolati

**Affiliations:** 1 College of Medicine, Ajman University, Ajman, ARE; 2 Department of Medical Imaging, American Hospital Dubai (AHD), Dubai, ARE; 3 Department of Radiology, Mediclinic Parkview Hospital, Dubai, ARE; 4 Department of Neuroscience, American Hospital Dubai (AHD), Dubai, ARE

**Keywords:** carotid cavernous fistula, ccf, cerebral angiography, contrast-enhanced mri angiography, dsa

## Abstract

A carotid-cavernous fistula (CCF) is an abnormal communication between the carotid artery and the cavernous sinus. Contrary to the typical insidious onset, we present a case of an indirect CCF in a 49-year-old woman who presented with the sudden onset of a painful, swollen, and red left eye, which was initially misdiagnosed as orbital cellulitis. It is crucial to maintain a high index of suspicion in all patients who present with the sudden onset of ocular symptoms, particularly if they exhibit pulsatile proptosis, chemosis with arterialized conjunctival veins, periorbital bruit, elevated intraocular pressure (IOP), and normal inflammatory markers. Early diagnosis is essential to prevent vision loss and other associated neurological complications. The patient underwent transvenous coil occlusion of the left cavernous sinus but later developed a recurrent fistula, underscoring the need for close follow-up after endovascular intervention to detect any residual or recurrent fistula.

## Introduction

Carotid-cavernous fistulas (CCFs), or caroticocavernous fistulae, are abnormal connections between the carotid artery and the cavernous sinus. They can be categorized as either direct or indirect types based on their etiology and vascular anatomy. Approximately 70%-75% of all CCFs are trauma-related, typically affecting young males, and are usually high-flow, direct fistulas. Patients often present with sudden ocular symptoms that can resemble inflammatory or allergic eye conditions. In contrast, spontaneous CCFs make up 25%-30% of cases and are linked to underlying vascular connective tissue diseases, which increase the risk of vascular injuries [1-3]. These usually cause low-flow indirect fistulas and are more commonly seen in older females. These fistulas tend to have a gradual onset and often present with more subtle ocular symptoms [1,4].

The clinical presentation of CCFs varies according to the type and hemodynamics of the fistula. Some cases may present with classic ocular symptoms, whilst others may present atypically, posing diagnostic challenges [5]. This case is particularly noteworthy due to the unusual, rapid onset of an indirect CCF, which initially resembled an eye infection, highlighting the diagnostic challenge in distinguishing it from common acute ocular conditions. Indirect CCFs, in particular, are low-flow fistulas and can either be asymptomatic or gradually progress with subtle symptoms. If left untreated, cranial nerve palsies, ophthalmoplegia, and other ocular complications may develop. 3D T1-weighted contrast-enhanced magnetic resonance imaging (MRI) angiography, with its high resolution capability, can suggest the diagnosis by demonstrating early venous filling. However, conventional cerebral angiography is the gold standard for definitive diagnosis, planning, mapping, and performing endovascular treatment [5-7].

Endovascular treatment is the primary management approach for CCFs, with transvenous and transarterial approaches demonstrating high success rates of up to 80% in obliterating the fistula [8,9]. This case demonstrates an unusual presentation of a CCF, highlighting the importance of early diagnosis as well as close clinical and imaging follow-up for possible recurrence.

This case was previously presented as a poster at the 11th Evolving Practice of Ophthalmology Middle East Conference on December 5-7, 2024.

## Case presentation

**Figure 1:**
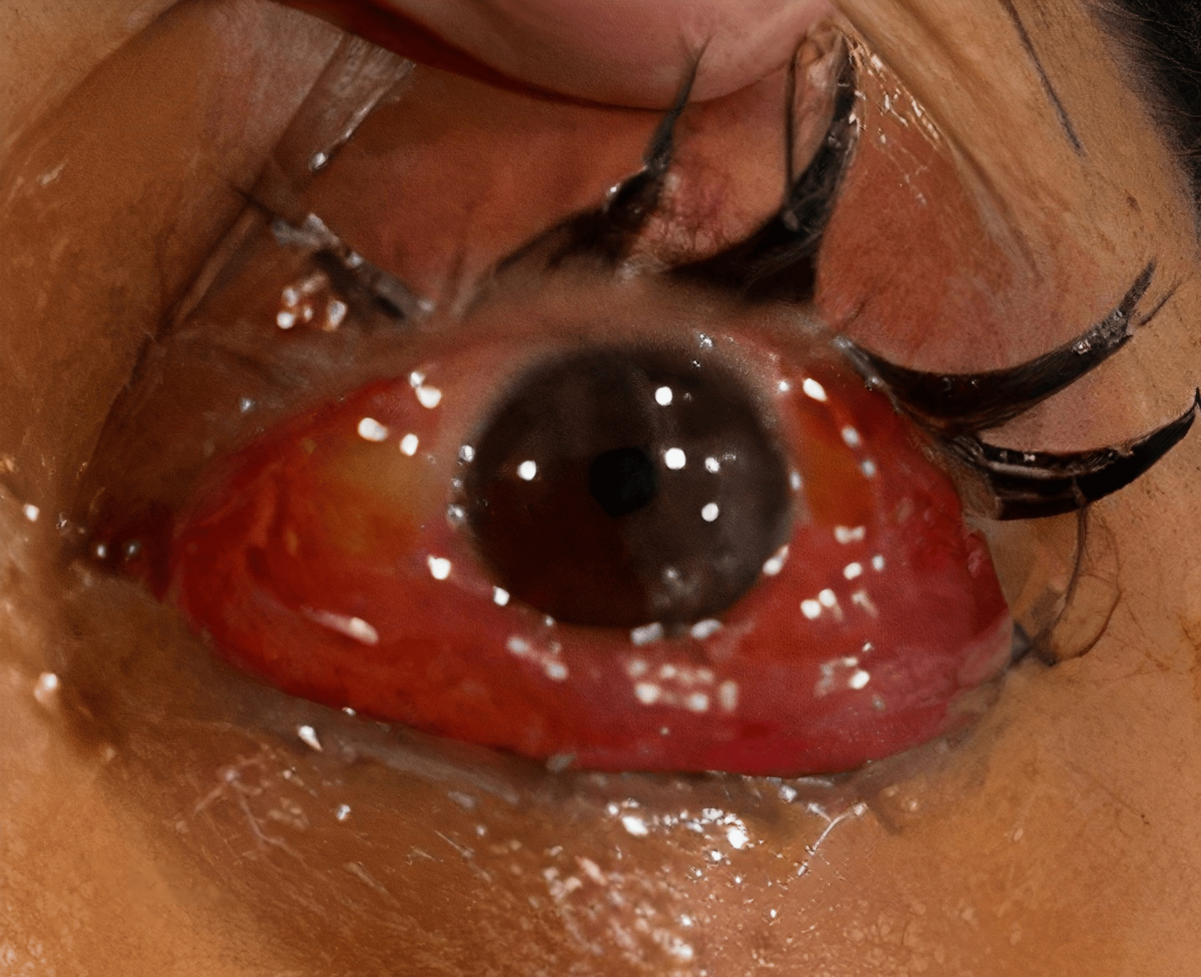
Left eye showing conjunctival erythema, chemosis, and proptosis.

A 49-year-old female presented to the emergency department with a history of a sudden onset of red, painful, and swollen left eye for the last two days. She had recently visited an ophthalmology clinic and was prescribed eye drops for suspected infection or allergy without improvement. Her past medical history included a recent upper respiratory tract infection, cosmetic surgery on the lip and nose a month prior, and rheumatoid arthritis managed with non-steroidal anti-inflammatory drugs. On examination, the left eye showed significant swelling of the upper and lower eyelids with conjunctival erythema and mild proptosis (Figure 1). The visual acuity in the left eye was reduced to 20/60 compared to 20/20 in the right eye. Both pupils were equal and reactive. Tonometry revealed an elevated intraocular pressure (IOP) in the left eye of 36 mmHg compared to 17 mmHg in the right eye. She was afebrile, and vital signs were within normal limits. Laboratory results showed normal leukocyte count and inflammatory markers. A provisional diagnosis of orbital cellulitis was made, and the patient was treated with empiric intravenous antibiotics (vancomycin and piperacillin-tazobactam) and oral analgesia (ketorolac).

**Figure 2:**
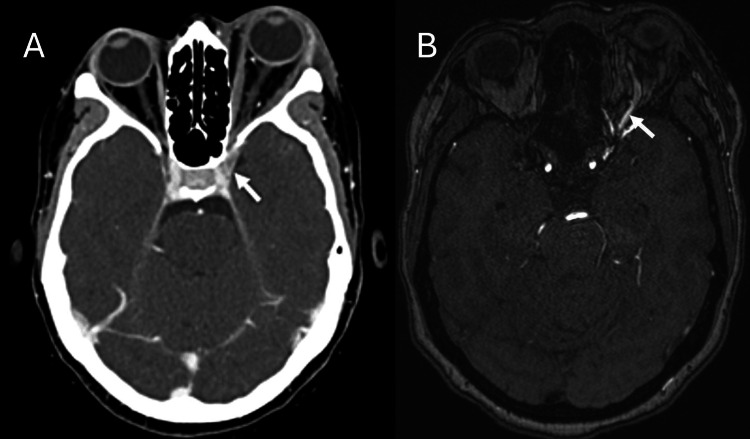
(A) Axial contrast-enhanced CT of the brain showing partial opacification and engorgement of the left cavernous sinus (arrow); (B) axial MRI angiography demonstrating early enhancement of the superior ophthalmic vein (arrow) in the arterial phase.

Contrast-enhanced computed tomography (CT) examination of the orbits demonstrated edema and congestion in the retro-orbital and periorbital soft tissues, but no abscess or fluid collection was identified. A significantly distended left superior ophthalmic vein with suboptimal enhancement of the engorged left cavernous sinus suggested partial cavernous sinus thrombosis (Figure 2A). All other venous sinuses were patent and showed normal enhancement. A contrast-enhanced MRI angiography revealed significant engorgement of the left superior ophthalmic vein with early filling during the arterial phase in keeping with CCF (Figure 2B). Consequently, clinical examination of the left eye revealed a bruit upon auscultation of the left periorbital region, supporting the diagnosis of CCF. This was further supported by the laboratory results of normal inflammatory markers.

**Figure 3:**
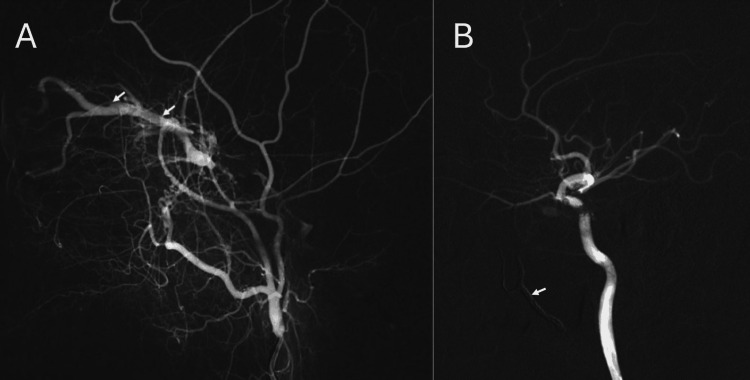
Digital subtraction angiography of the left carotid. (A) Pre-coiling image showing early filling of the engorged superior ophthalmic vein (arrows). (B) Post-coiling image demonstrating complete occlusion of the fistulous communications and absence of venous and cavernous sinus filling, achieved using embolization coils (arrow).

**Figure 4:**
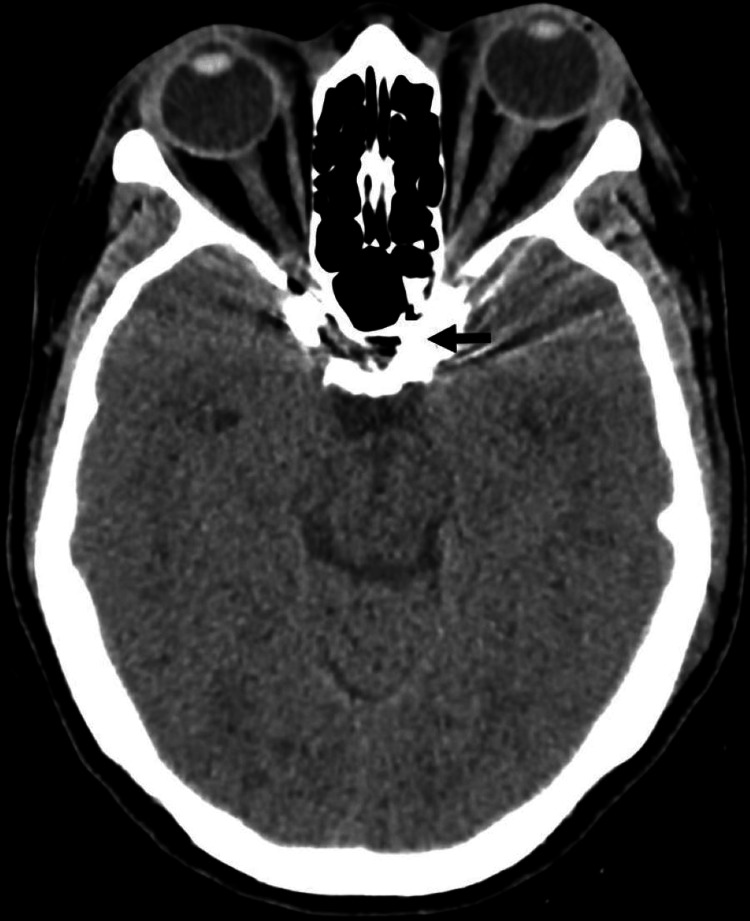
Axial non-contrast CT brain showing coils in the region of the left cavernous sinus (arrow) and no post-procedure complications.

**Figure 5:**
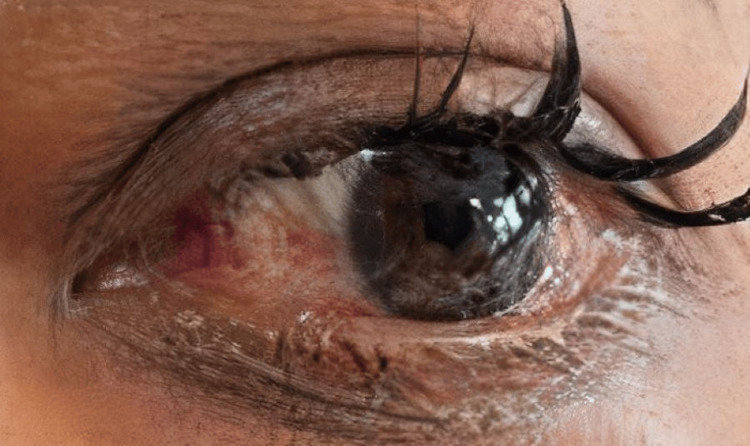
Post-endovascular treatment: appearances of the left eye showing significant reduction in redness, chemosis, and proptosis.

Digital subtraction angiography (DSA) confirmed a left type D CCF fed by branches of both the internal and external carotid arteries, with significant engorgement of the cavernous sinus and ophthalmic vein (Figure 3A). The patient underwent successful transvenous coil occlusion of the left cavernous sinus, achieving complete obliteration of the fistulous communications (Figure 3B). Post procedure, the patient remained neurologically stable, and CT showed no complications (Figure 4). The patient showed a significant improvement in eye movement, vision, and reduction in the swelling, redness, chemosis, and proptosis (Figure 5). The left periorbital bruit was no longer present, and the patient was discharged with a three-week follow-up appointment.

**Figure 6:**
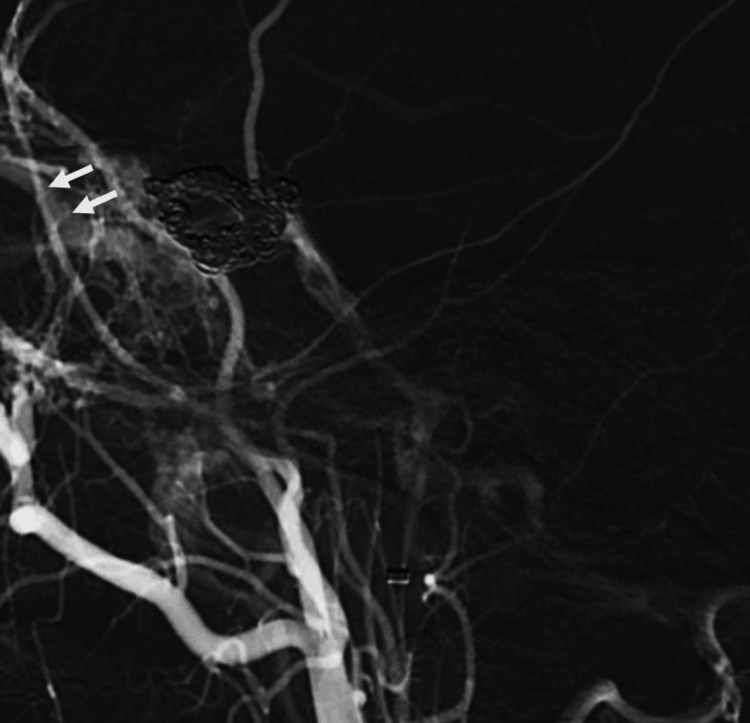
Follow-up digital subtraction angiography of the left carotid showing filling of the superior ophthalmic vein (arrows), indicative of a recurrent fistula.

At follow-up, the patient reported a slight increase in left eye swelling and redness for the past two days, particularly first thing in the morning. Clinical examination revealed maintained visual acuity and fields. While the IOP in the right eye remained normal at 14 mmHg, the left eye showed a significant reduction from 36 to 24 mmHg. A subtle bruit was noted on auscultation of the left periorbital region. A follow-up cerebral angiography was performed two weeks later, which confirmed the presence of a recurrent left type D CCF (Figure 6), necessitating another endovascular coil occlusion of the residual carotid artery branches. The patient recovered well with normal eye examination findings apart from minor residual conjunctival congestion.

## Discussion

A CCF is an abnormal communication between the carotid artery and the cavernous sinus. CCFs are typically classified as direct (type A) and indirect (types B-D). Type A is caused by direct communication between the cavernous segment of the internal carotid artery and the cavernous sinus. They are more common in men and are usually caused by trauma or, less commonly, following surgical or endovascular procedures [1,2]. The indirect types are usually seen in postmenopausal women with a 7:1 female-to-male ratio. Indirect CCFs are usually spontaneous and result from abnormal communication between the cavernous sinus and extradural branches of the internal carotid artery (type B), external carotid artery (type C), or both (type D) [2,4]. These indirect fistulas are commonly associated with hypertension, atherosclerosis, and pregnancy. Additionally, collagen vascular diseases have also been implicated as potential risk factors [10].

The majority of the cases of CCFs occur following head trauma. The incidence of CCF is not well documented; however, CCFs are seen in 0.2% of patients with traumatic brain injury and up to 4% of patients with a base of skull fracture [5,6]. CCFs are usually unilateral, but they can be bilateral in up to 1% of patients with traumatic brain injuries [11].

The clinical presentation of CCF depends on etiology, type, size, flow rate, and location of the fistula. The earliest and most common symptoms are ocular symptoms and are linked to disruptions in the venous drainage from the orbits to the cavernous sinuses. Ophthalmic signs include eyelid edema, ptosis, conjunctival arterialization, elevated IOP, and pulsatile proptosis. Ophthalmic complications may also occur, such as pupillary abnormalities, papilledema, ischemic optic neuropathy, central retinal vein occlusion, or choroidal detachment [1]. Direct CCF is a high-flow fistula and usually presents with a sudden onset of symptoms; however, indirect CCFs are low-flow fistulas and may be asymptomatic or have an insidious progressive course. Alexander et al found that the majority of patients with indirect CCF have a mean duration from symptom onset to diagnosis of 234 days [12]. Cranial nerve palsies involving the third, fourth, and sixth nerves, as well as ophthalmoplegia, can occur in up to 63% of cases. Horner's syndrome and sixth nerve palsy may occur simultaneously or sequentially due to compression within the cavernous sinus [4,13].

The initial non-invasive workup usually consists of contrast-enhanced CT or MRI angiography to delineate early filling of the dilated superior ophthalmic vein during the arterial phase. Contrast-enhanced MRI angiography has distinct advantages over other imaging modalities, including the capability to perform multiphasic imaging similar to DSA, without exposure to ionizing radiation. Currently, DSA remains the gold standard for diagnosing CCFs, as it is essential for confirming the diagnosis and planning and guiding therapeutic interventions [14-16].

Management options for CCF include conservative treatment, open surgery, stereotactic radiosurgery, conventional radiotherapy, and endovascular intervention via an arterial or venous route, with the latter being the most widely utilized approach. Treatment aims to maintain blood flow in the internal carotid artery as well as occluding the fistulous communications. Indirect CCFs may undergo spontaneous closure in 20%-60% of cases, and therefore, conservative management should be considered in relatively asymptomatic patients with low flow. Direct CCFs, as previously indicated, are usually high flow and invariably require intervention [8,15,16]. Recurrence is a recognized complication of CCF, as in our case; therefore, close clinical monitoring and follow-up imaging are crucial [17]. The success rate of endovascular intervention is over 80%; however, it has a recurrence rate of up to 28% [9].

## Conclusions

Contrary to its typical insidious presentation, indirect CCF may present with a sudden onset of ocular symptoms, as typically seen in type A direct CCF. A high level of clinical suspicion of CCF is important in all patients presenting with an acute, painful, swollen eye, even in the absence of recent trauma. Inflammatory eye conditions such as orbital cellulitis can present in this manner; however, CCF can be suspected in the presence of the following signs: pulsatile proptosis, chemosis with arterialization of the conjunctival veins, periorbital bruit, raised IOP, normal inflammatory markers, and lack of clinical improvement with antibiotic treatment. Contrast-enhanced MRI angiography can suggest the diagnosis by demonstrating the typical early filling of the engorged superior ophthalmic vein during the arterial phase. Early diagnosis is of paramount importance in managing this potentially vision-impairing condition and minimizing possible neurological complications. Conventional cerebral angiography is the mainstay for confirming diagnosis, providing endovascular treatment, and subsequent follow-up.
